# Signal-transducing adaptor protein-2 promotes generation of functional long-term memory CD8^+^ T cells by preventing terminal effector differentiation

**DOI:** 10.18632/oncotarget.15403

**Published:** 2017-02-16

**Authors:** Daisuke Muraoka, Naohiro Seo, Tae Hayashi, Chisaki Hyuga-Amaike, Kana Okamori, Isao Tawara, Naozumi Harada, Hiroshi Shiku

**Affiliations:** ^1^ Department of Immuno-Gene Therapy, Mie University Graduate School of Medicine, Mie, Japan; ^2^ Center for Drug Discovery, Graduate School of Pharmaceutical Sciences, University of Shizuoka, Shizuoka, Japan; ^3^ Department of Hematology and Oncology, Mie University Graduate School of Medicine, Mie, Japan

**Keywords:** memory T cell, CTL function, STAP2, STAT signaling, SOCS3, Immunology and Microbiology Section, Immune response, Immunity

## Abstract

Long-surviving memory CD8^+^ T cells generated by stimulation with appropriate tumor-associated antigens are the most aggressive and persistent tumoricidal effectors. In this event of memory CD8^+^ T cell development, the signal transducer and activator of transcription (STAT) proteins function as the crucial intracellular signaling molecules, but the regulatory mechanism of STATs in CD8^+^ T cells is not fully understood. In this study, we report for the first time, by using murine vaccination models, that signal-transducing adaptor protein-2 (STAP2) maintains the cytotoxicity of long-lived memory CD8^+^ T cells by controlling a STAT3/suppressor of cytokine signaling 3 (SOCS3) cascade. Following T cell activation, STAP2 expression was transiently reduced but was subsequently recovered and augmented. Analysis using small-interfering RNA (siRNA) demonstrated that restored STAP2 expression was associated with the activation of STAT3/SOCS3 signals and maintenance of cytotoxic T lymphocytes (CTLs) secondary responses by preventing their differentiation into terminal effector cells. Notably, this STAP2-dependent memory differentiation was observed in the spleen, but not in the lymph nodes (LNs). These findings indicate an essential role for STAP2 in the generation of a high-quality memory CD8^+^ CTLs periphery, and suggest the therapeutic potential of STAP2 in cancer patients.

## INTRODUCTION

Memory CD8^+^ CTLs play a central role in infection prophylaxis and surveillance against malignancy [1, 2]. When naive CD8^+^ T cells encounter microorganisms or tumor-derived antigens, they proliferate extensively and differentiate into the effector phenotype accompanied with vigorous cytokine production and strong catalytic ability. Most of them eventually undergo apoptosis a little while after differentiation into interleukin (IL)-7^−^ effector T cells. [3–5] However, a small portion (5-10%) of these differentiates into the IL-7R^+^ memory phenotype, classified into stem cell memory T (Tscm) cells, central memory T (Tcm) cells, and effector memory T (Tem) cells on the basis of cell surface markers, proliferation activity, and cytotoxicity. [6–8] Memory T cells exhibit persistent growth following rapid response to antigen reencounter compared with temporal effector T cells, suggesting the importance of elucidating the molecular mechanisms of memory T cell differentiation in the development of immunotherapy with prolonged tumoricidal effects [7–9].

Formation of memory CD8^+^ T cells is controlled by several factors such as antigen stimulation, cytokines, transcription factors, and metabolism [8, 10–12]. Specific cytokine signals regulate CD8^+^ T cell differentiation and memory formation as follows: IL-7 and IL-15 promote CD8^+^ memory T cell survival and self-renewal [13], IL-2 controls the balance of memory and effector T cell differentiation by regulating eomesodermin (Eomes) expression [5], and IL-10 and IL-21 signals are crucial for the formation of mature self-renewing memory T cells in a B cell lymphoma-6-mediated manner [14].

STATs play an essential role in the regulation of CD8^+^ memory T cell differentiation via cytokine signaling and cytokine production in antigen-stimulated CD8^+^ T cells. For example, during memory T cell differentiation, STAT5 or STAT3 is required for signaling via IL-2, IL-7, and IL-15, or IL-10 and IL-21, respectively [5], [14–16], SOCS family proteins act as an intracellular regulator of STAT signals. IL-12/STAT4 signal-mediated induction of SOCS5 inhibits STAT6 signals, resulting in suppression of Th2 cell development, whereas, SOCS3 induced by IL-4/STAT6 signals downregulates Th1 differentiation by modulating IL-12/STAT4 signals. [17], [18] Inhibition of IL-12/STAT4 signals by SOCS3 promotes functional memory CD8^+^ T cell maturation. However, the molecular mechanisms regulating these signals by intracellular molecules such as adaptor proteins remain to be elucidated.

STAP2 is a signal adaptor protein that contains an N-terminal pleckstrin homology (PH) domain and a region that is distantly related to the Src homology 2 (SH2) domain. The PH and SH2 domains, and the YXXQ motifs in STAP2 bind to focal adhesion kinase, IκB kinases, myeloid differentiation primary response gene 88, STAT5, latent infection membrane protein 1, and STAT3. [19–22] Previous studies have shown that STAP2 attenuates IL-2-dependent and TCR-mediated proliferation of thymocytes and enhances stromal cell-derived factor-1α-induced T cell migration. [22, 23] STAP2 has also been reported to induce strong immune responses to lipopolysaccharide or inflammatory cytokines such as IL-6 in hepatocytes due to enhanced IL-6/STAT3 signaling. [19]

In this study, we demonstrated that STAP2 is preferentially expressed in memory T cells and acts as a novel regulator participating in CD8^+^ T cell memory differentiation without inducing a terminal effector phenotype. In addition, we concluded that STAP2 is essential for the maintenance of long-term memory CTL function through IL-10- and IL-21-mediated modifications of the STAT3/SOCS3 pathway.

## RESULTS

### Identification of STAP2 as a candidate regulator in the differentiation of vaccine-induced memory CD8^+^ T cells

**Figure 1:**
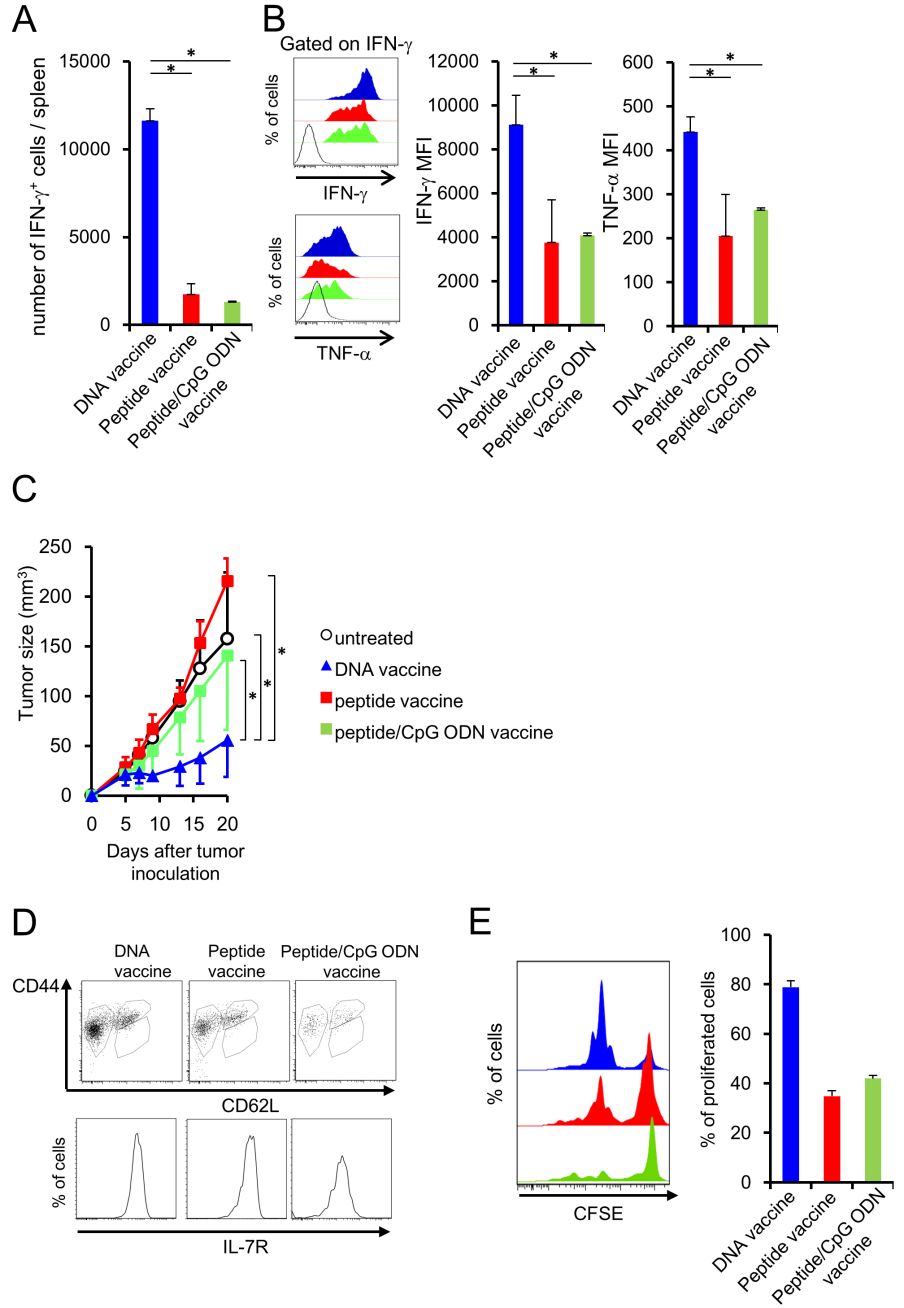
DNA vaccination induces the functional memory CD8^+^T cells BALB/c mice were injected with NY-ESO-1_81-88_ peptide emulsified in IFA with or without CpG ODN or plasmids encoding the entire NY-ESO-1 protein, using a gene gun. **A**., **B**. At 44 days after the last immunization, CT26-NY-ESO-1 was inoculated into the immunized mice. At 14 days after tumor inoculation, splenocytes from the mice were incubated with NY-ESO-1_81-88_ or control peptide, and cytokine secretion was analyzed by flow cytometry. Production of TNF-α and IFN-γ was negligible, when the splenocytes were stimulated with the control peptide. (Blue filled; DNA vaccine, Red filled; peptide vaccine, black line; isotype control). **C**. Tumor sizes were monitored twice or thrice per week (n = 5/group). **D**. At 44 days after the last immunization, the expression of CD44, CD62L and IL-7R on vaccine-specific CTLs cell was analyzed by flow cytometer. **E.** At 44 days after the last immunization, splenocytes were labeled with CFSE and stimulated with mERK2 peptide for 3 days and proliferation activity was analyzed by flow cytometer.

DNA, peptide and peptide/adjuvant vaccinations were performed to induce memory T cell differentiation. BALB/c mice were injected with NY-ESO-1_81-88_ peptide (RGPESRLL: an H-2D^d^-restricted murine CTL epitope of human NY-ESO-1) with or without CpG ODN as adjuvant, or plasmid DNA encoding the whole NY-ESO-1 sequence using a gene gun. [24] At 44 days after the last immunization, mice were subcutaneously inoculated with CT26-NY-ESO-1 cells. [25] Splenocytes were isolated at 14 days after tumor inoculation and incubated with NY-ESO-1_81-88_ peptide or the control peptide to assess cytokine secretion. The number of interferon (IFN)-γ-producing NY-ESO-1_81-88_-specific CD8^+^ T cells and the magnitude of IFN-γ and tumor necrosis factor (TNF)-α secretion in individual NY-ESO-1_81-88_-specific CD8^+^ T cells was higher in the DNA vaccination group than in the peptide or peptide/CpG ODN vaccination group (Figure 1A and 1B), indicating the high efficacy of peptide-specific CD8^+^ T cell induction by DNA vaccination. Consistent with cytokine production, attenuation of subcutaneous CT26-NY-ESO-1 growth was observed in the DNA-vaccinated group more vigorously than in the peptide-vaccinated or peptide/CpG ODN-vaccinated group (Figure 1C). To address T cell activation state in each vaccinated mice, vaccination was performed by using the mice infused with CD8^+^ T cells from mutated (m) ERK2 tumor antigen-specific T cell receptor (TCR) gene transgenic DUC18 mice. [26, 27] As indicated in Figure 1D and 1E, all vaccines differentiated the infused T cells into central memory (CD62L^−^ CD44^+^ IL-7R^+^) or effector memory (CD62L^+^ CD44^+^ IL-7R^+^) state. However, intense proliferation of the infused T cells was shown in only DNA vaccination, but not peptide or peptide/CpG ODN vaccination. These results indicate the effective generation of functional memory T cells by DNA vaccination compared with peptide or peptide/CpG ODN vaccination.

**Figure 2:**
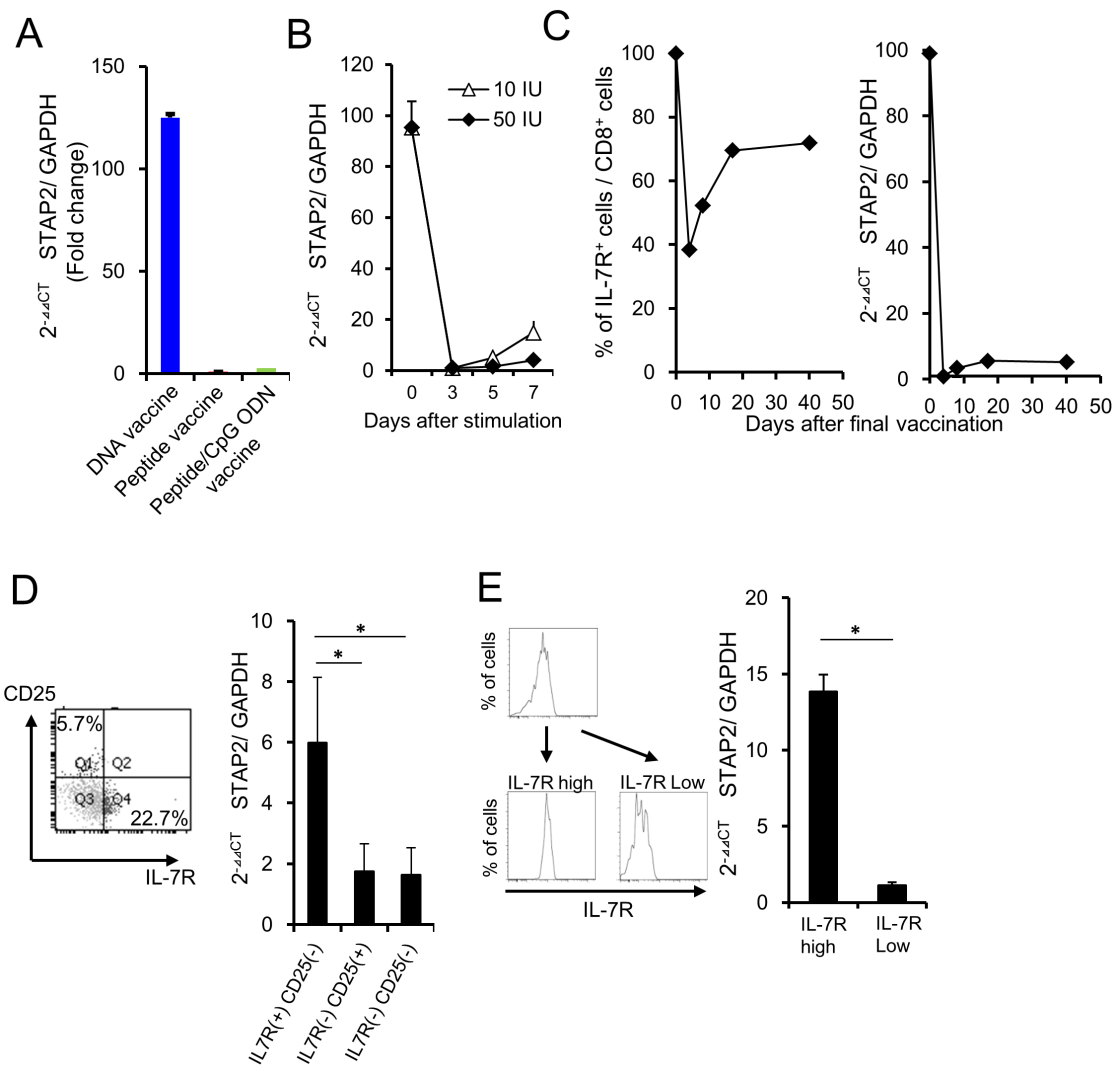
STAP2 regulates vaccine-induced memory CD8^+^ T cell differentiation **A**. At 7 days after the last vaccination, vaccine-specific CTLs were collected from draining the LNs, and RT-PCR was performed to quantify STAP2 mRNA levels. **B**. Splenocytes derived from DUC18 mice were stimulated with mERK2 peptide for 3 days and then cultured in rIL-2 (10 or 50 IU/ml)-containing medium for 4 days. STAP2 expression in cultured cells was measured by RT-PCR. **C**. CD8^+^ T cells derived from DUC 18 mice were infused in BALB/c mice. Mice were then vaccinated with mERK2-coding plasmid DNA and infused cells were collected on the indicated days. STAP2 expression was measured by RT-PCR. **D**. On d-7 after antigen stimulation, IL-7R positive and negative cells were sorted, and analyzed by RT-PCR for STAP2 expression. **E**. On day 7 after the last vaccination, IL-7R positive and negative cells were sorted from the infused mice and analyzed by RT-PCR for STAP2 expression. The results are representative of two to four experiments. Data are expressed as the mean ± SD. *p < 0.05 is considered significant.

To identify the novel molecules involved in functional memory T cell differentiation, mERK2 peptide-specific memory precursor T cells from the vaccinated mice were collected and subjected to microarray analysis (Expression of the memory T cell-related genes was not different between DNA vaccine and peptide vaccine or peptide/CpG vaccine (-1<Log2 ratio<1) [Table 1]). We focused on cytoplasmic molecules that control the STAT signaling, because STATs are known as representative molecules involved in memory T cell differentiation (Table 2). We selected STAP2, an adaptor protein of STATs, as a novel candidate participating in memory T cell differentiation because of its strong expression in the memory precursors from the DNA-vaccinated group but not peptide-vaccinated groups (Figure 2A). [19–22] By monitoring of STAP2 expression in mERK2 peptide-specific CD8^+^ T cells after vaccination, we clarified that STAP2 expression was temporarily downregulated, and gradually restored and increased thereafter (Figure 2B). In addition, sorted IL-7R^+^ CD8^+^ T cells expressed higher levels of STAP2 than the IL-7R^−^ CD8^+^ T cell population consistent with the results from different vaccination models (Figure 2C-2E), indicating the superiority of STAP2 expression in memory CD8^+^ T cells. Additionally, we measured the STAP2 expression in IL-7R^+^ KLRG1^−^ and IL-7R^+^ KLRG1^+^ cells, and concluded that the expression of STAP2 is higher in IL-7R^+^ KLRG1^+^ and IL-7R^+^ KLRG1^−^ populations than that in IL-7R^−^ KLRG1^−^ and IL-7R^+^ KLRG1^+^ populations (Supplementary Figure S1), likely with Figure 2C.

**Table 1:** The expression of genes related to memory T cells was not different among the DNA vaccination, peptide vaccination, and peptide/CpG ODN vaccination groups

Probe Set ID	Gene Symbol	Log2 Ratio *vs* Peptide Vaccine	Log2 Ratio *vs* Peptide/CpG ODN Vaccine
1419481_at	selectin, lymphocyte	0.41	0.86
1448575_at	interleukin 7 receptor	-0.49	-0.62
1426191_a_at	BCL2-like 1	0.17	0.28
1452389_at	CD27 antigen	-0.08	0.01

**Table 2:** Comparison of gene expression in memory precursor T cells in the DNA vaccination group with that in the peptide vaccination and peptide/CpG ODN vaccination groups

Probe Set ID	Gene Symbol	Log2 Ratio *vs* Peptide Vaccine	Log2 Ratio *vs* Peptide/CpG ODN Vaccine
1448664_a_at	Speg	-6.13	-6.88
1442593_at	---	-2.91	-6.45
1435884_at	Itsn1	-3.38	-5.69
1436814_at	---	-2.10	-5.62
1437800_at	Edaradd	-6.54	-5.37
1453683_a_at	Cep55	-1.98	-5.22
1416675_s_at	Plcd1	-3.18	-5.13
1459714_at	---	-4.59	-4.99
**1424148_a_at**	**Stap2**	**-1.68**	**-4.95**
1424254_at	Ifitm1	-2.84	-4.93
1440728_at	---	-1.65	-4.91
1449991_at	Cd244	-2.48	-4.65
1458625_at	---	-4.02	-4.65
1453277_at	3021401N23Rik	-2.03	-4.57
1417749_a_at	Tjp1	-1.93	-4.54
1446045_at	---	-1.97	-4.44
1444853_at	---	-2.49	-4.34
1441476_at	Socs2	-1.99	-4.22
1436470_at	Rims2	-2.57	-4.20
1442913_at	---	-3.70	-4.02

### Necessity of STAP2 for the maintenance of CTL function and the prevention of CD8^+^ T cell differentiation into terminal effector

**Figure 3:**
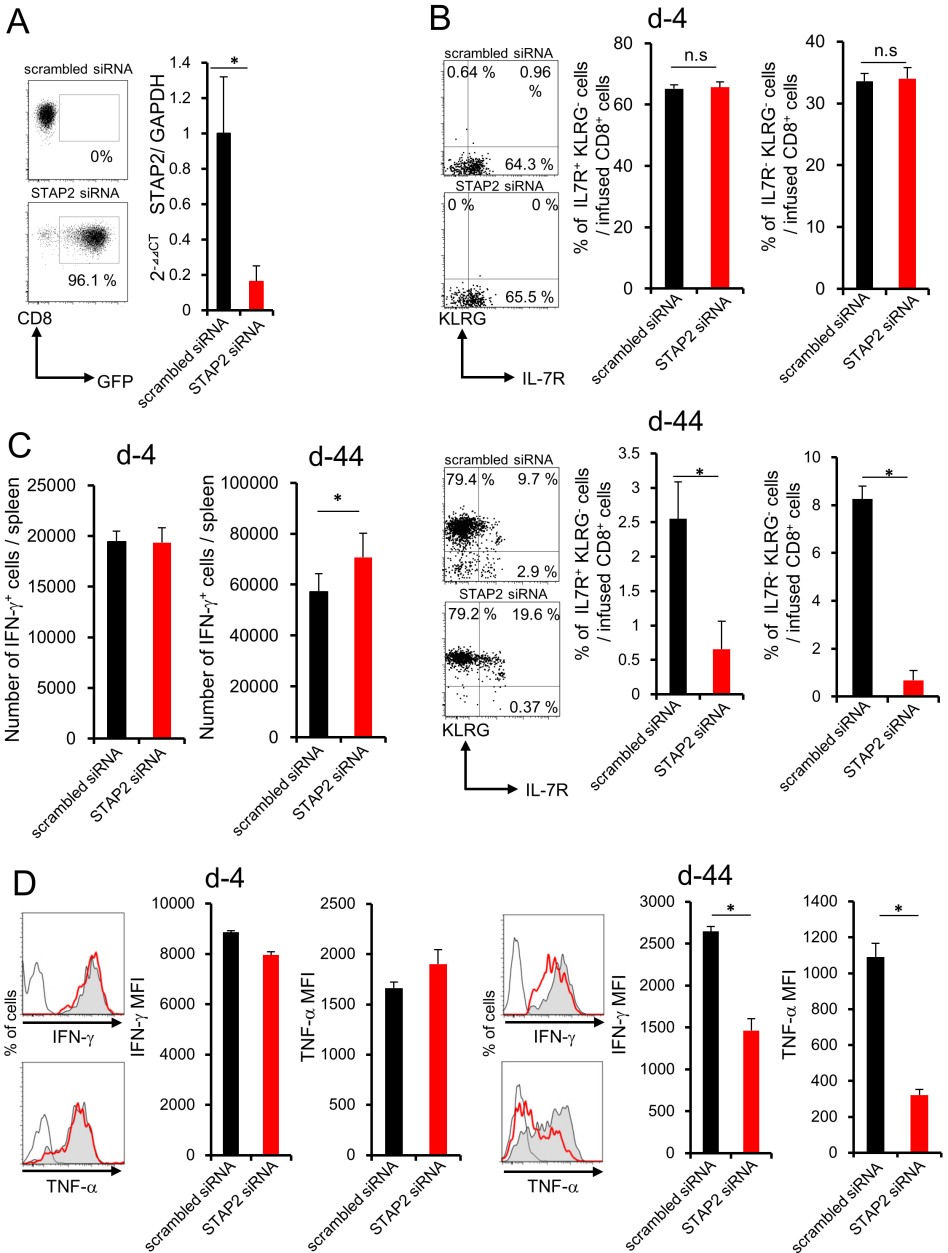
STAP2 has a crucial role in memory generation but not in effector cell differentiation in the spleen Splenocytes derived from DUC18 mice were stimulated with mERK2 peptide for 3 days, transduced with STAP2-specific siRNA, scrambled control siRNA, or GFP mRNA by electroporation, and injected into BALB/c mice. **A**. Transfection efficacy of GFP mRNA and knockdown efficacies of STAP2 mRNA were examined by flow cytometric analysis and qRT-PCR, respectively. At 4 or 44 days after infusion, **B**., **C**., **D**. the number of IFN-γ^+^ T cells, the expression of memory markers (KLRG and IL-7R), and IFN-γ and TNF-α production were examined. (Gray filled; scrambled siRNA, Red line; STAP2 siRNA, black line; non-immunized control). The results are representative of two to four experiments. Data are expressed as the mean ± SD. *p < 0.05 is considered significant.

siRNA was used for silencing STAP2 mRNA to investigate whether STAP2 is required for the maintenance and generation of memory T cells. STAP2 mRNA in mERK2 peptide-stimulated DUC18 CD8^+^ T cells was effectively knocked down by transfection with STAP2-specific siRNA compared with scrambled control siRNA on the secured transfection efficacy by assessing green fluorescent protein (GFP) mRNA (Figure 3A). At 4 or 44 days after infusion, total number of the infused cells did not change by STAP2 KD in the spleen and dLN (Supplementary Figure S2). However, the KLRG^−^ IL-7R^+^ memory or KLRG^−^ IL-7R^−^ memory precursor population was significantly reduced in the spleen of STAP2 KD DUC18 CD8^+^ T cell-infused BALB/c mice compared with control mice on d-44, but not d-4, post infusion (Figure 3B). [3, 28] In addition, while increase in the number of IFN-γ^+^ CD8^+^ T cells was seen in the splenocytes of STAP2 KD DUC18 T cell-infused mice, IFN-γ and TNF-α secretions were downregulated in correlation with acquisition of KLRG expression on d-44, but not on d-4, post infusion, likely with a naïve DUC18 CD8^+^ T cell-using case (Figure 3C and 3D). Thus, STAP2 prevents KLRG^+^ terminal differentiation of effector cells in the maintenance and generation of splenic memory CTL function following antigen stimulation.

**Figure 4:**
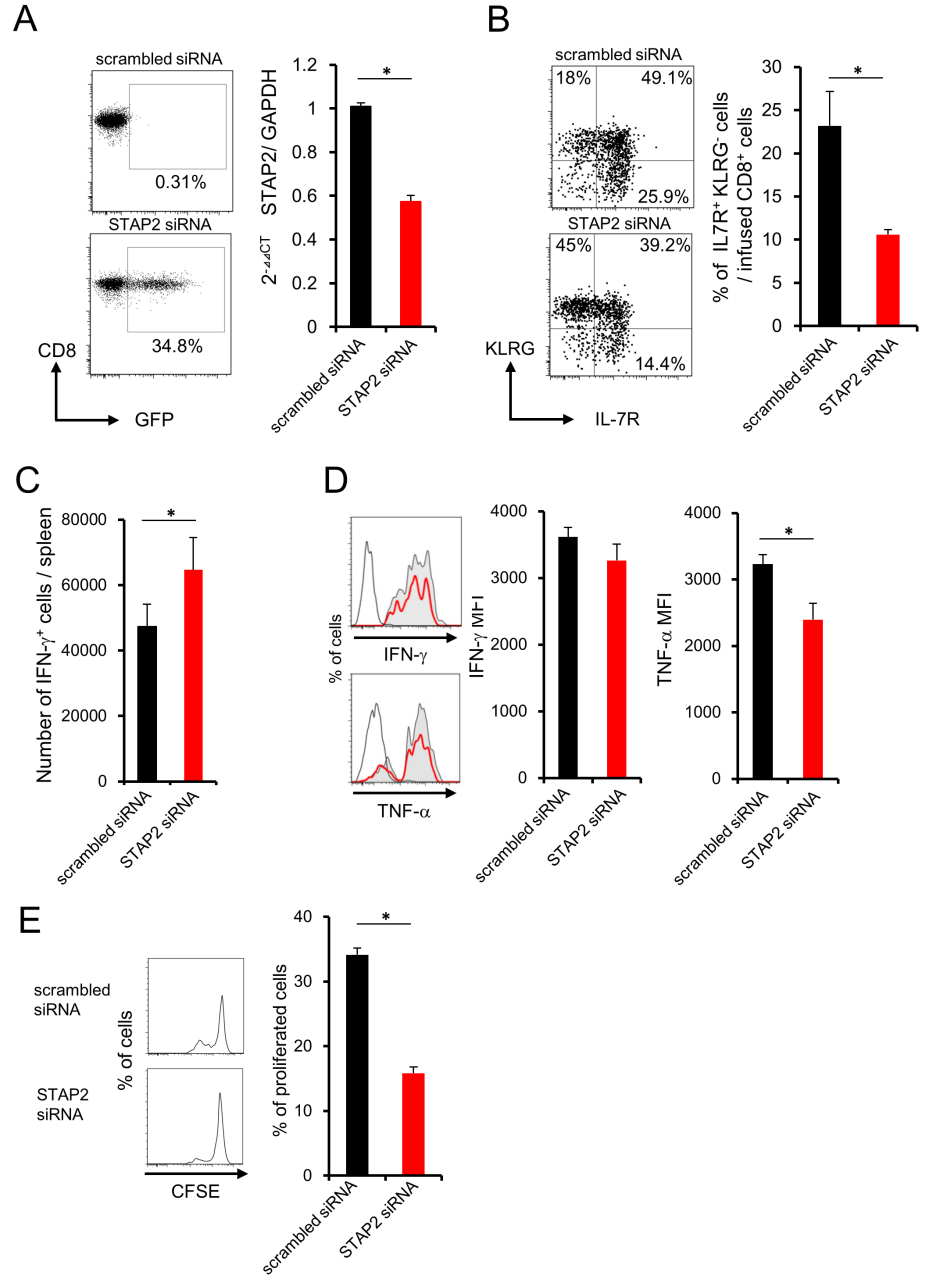
Importance of STAP2 on the elicitation of functional CD8^+^ memory T cells by ***in vivo*** priming Naïve CD8^+^ cells derived from DUC18 mice were transduced with STAP2-specific siRNA, scrambled control siRNA, or with GFP mRNA by electroporation, and injected into BALB/c mice. These BALB/c mice were then immunized with plasmids encoding mERK2 using a gene gun. **A**. At 2 days after transfection, expression of GFP and the knockdown efficacy of STAP2 mRNA was examined by flow cytometric analysis and qRT-PCR, respectively. **B**., **C**., **D**., **E**. At 44 days after vaccination, the number of IFN-γ^+^ T cells, the expression of memory markers (KLRG and IL-7R), the production of IFN-γ and TNF-α and proliferation were examined. (Gray filled; scrambled siRNA, Red line; STAP2 siRNA, black line; non-immunized control). The results are representative of two to four experiments. Data are expressed as the mean ± SD. *p < 0.05 is considered significant.

We further investigated the crucial role of STAP2 in memory generation by using *in vivo* vaccination system. STAP2 mRNA expression was disrupted in DUC18 splenic naïve CD8^+^ T cells by introduction of specific siRNA (Figure 4A), and then infused into BALB/c mice. At 44 days after vaccination with mERK2-coded DNA in the infused mice, reduced KLRG^−^ IL-7R^+^ and increased IFN-γ^+^ CD8^+^ T cell numbers were observed in the spleen of STAP2 KD naïve CD8^+^ T cell-transferred mice compared with the control mice (Figure 4B and 4C). In this model, TNF-α expression of CD8^+^ T cells was attenuated (Figure 4D). Notably, CD8^+^ T cells from STAP2 siRNA-treated group exhibited reduced proliferation capacity after DNA vaccination (Figure 4E). Taken together, STAP2 acts in the maintenance and generation of splenic memory CTLs by preventing *in vivo* CD8^+^ T cell differentiation into terminal effector.

**Figure 5:**
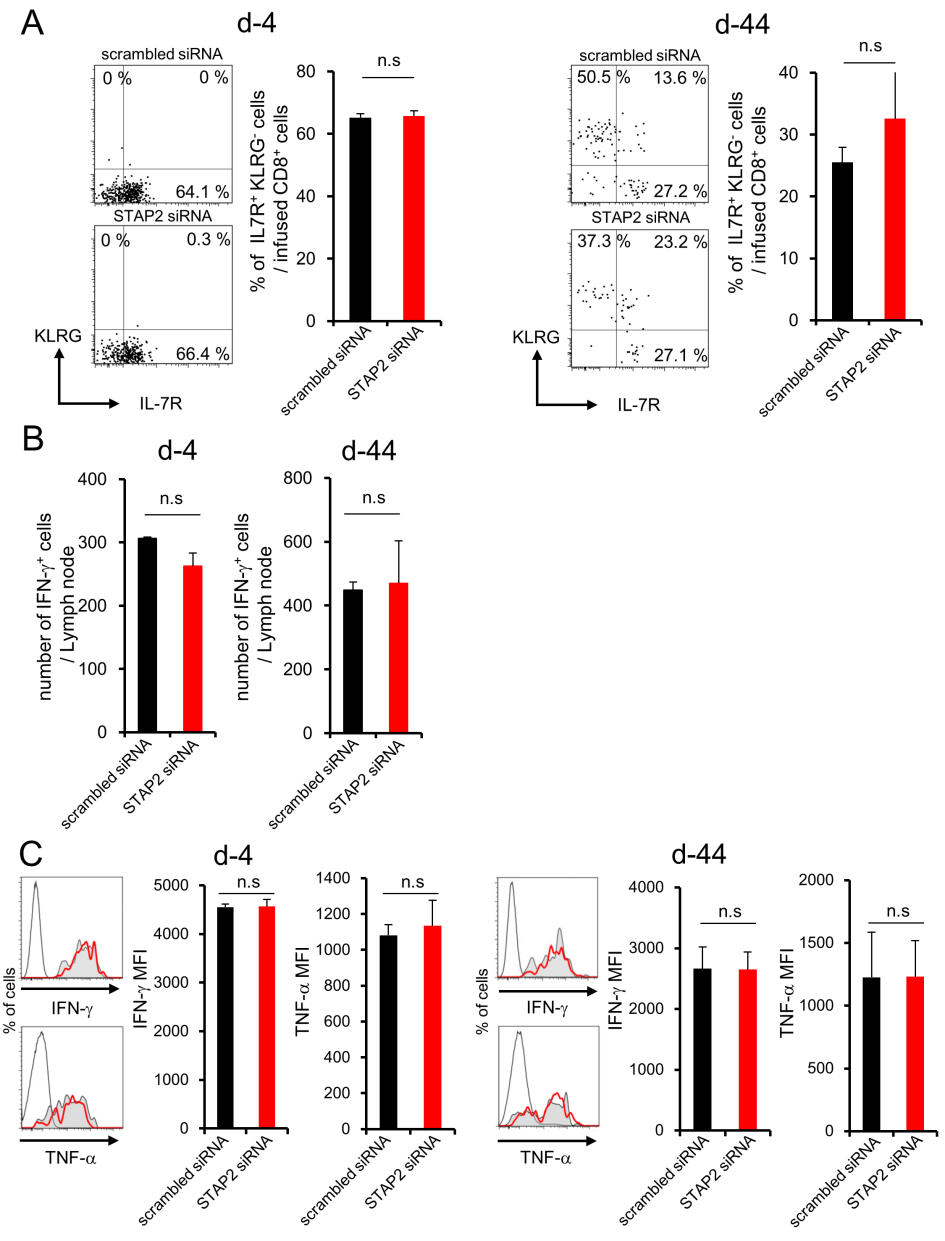
STAP2 has no capacity for memory CD8^+^ T cell differentiation in the LN Splenocytes derived from DUC18 mice were stimulated with mERK2 peptide for 3 days, transduced with STAP2-specific or scrambled control siRNA by electroporation, and then infused into BALB/c mice. At 4 or 44 days after infusion, these cells were collected from LNs and examined for the number of IFN-γ^+^ T cells, expression of memory markers (KLRG and IL-7R), and IFN-γ and TNF-α production by flow cytometry. (Gray filled; scrambled siRNA, Red line; STAP2 siRNA, black line; non-immunized control). The results are representative of two to four experiments. Data are expressed as the mean ± SD. *p < 0.05 is considered significant. n.s. = not significant.

Surprisingly, fluctuation in KLRG, IL-7R, IFN-γ, or TNF-α expression was not observed in CD8^+^ T cells of LNs (Figure 5A-5C).

### Maintenance of secondary responses of anti-tumor memory CD8^+^ T cells by STAP2

**Figure 6:**
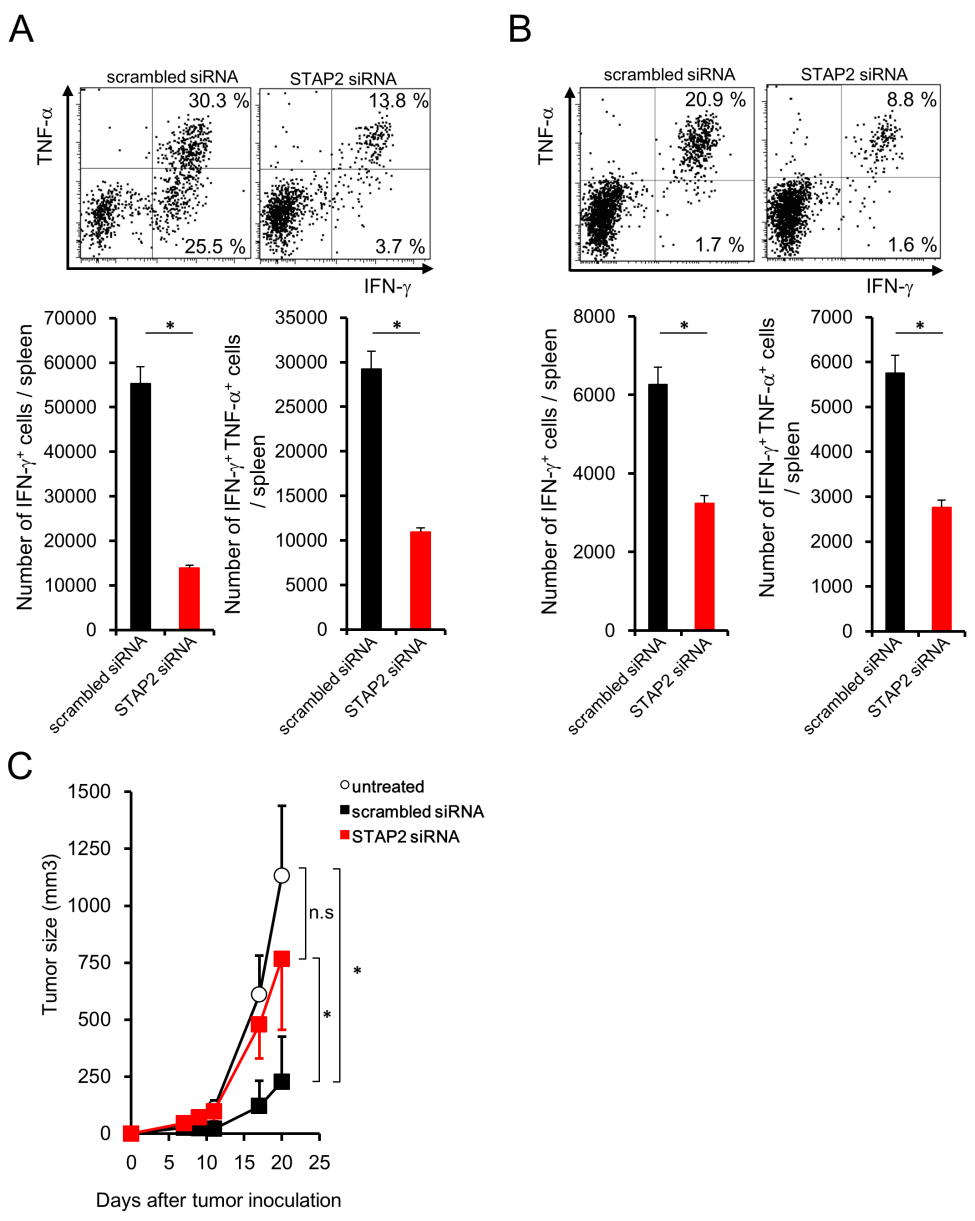
STAP2-mediated augmentation of secondary memory CD8^+^ T cell responses **A**. Splenocytes derived from DUC18 mice were stimulated with mERK2 peptide for 3 days, transduced with STAP2-specific or scrambled control siRNA by electroporation, and infused into BALB/c mice. At 44 days after infusion, CMS5a cells were subcutaneously inoculated into these mice and then collected CD8^+^ T cells were subjected to cytokine production assay. **B**. BALB/c mice were infused with siRNA-transduced DUC18 CD8^+^ cells and then immunized with plasmids encoding the mERK2 by gene gun. At 44 days after the final immunization, CMS5a were subcutaneously injected into these mice and splenic IFN-γ and TNF-α production was measured. **C**. CMS5a growth was monitored in (A) (*n* = 5/group). The results are representative of two to four experiments. Data are expressed as the mean ± SD. *p < 0.05 is considered significant.

Fluctuation of the number of antigen-specific IFN-γ^+^ CD8^+^ T cells was very weak after priming regardless of STAP2 KD as shown in Figure 3C and 4C, suggesting the involvement of STAP2 on the maintenance of antitumor effects. Subcutaneous CMS5a tumor cell (mERK2^+^ fibrosarcoma line [BALB/c background])-bearing BALB/c mice were infused with STAP2 siRNA- or scrambled siRNA-introduced DUC18 CD8^+^ T cells, and then subjected to *in vitro* or *in vivo* vaccination with mERK2 peptide or mERK2-encoded DNA. Although no change of the infused cell number was shown (Supplementary Figure S3), the proportion of IFN-γ- and TNF-α-producing CD8^+^ T cells was significantly decreased in the STAP2 siRNA-treated group (Figure 6A and 6B), suggesting a correlation between reduced tumoricidal effects and STAP2 KD. In fact, attenuation of tumor growth was significantly observed in the scrambled siRNA-treated control group, but not the STAP2 siRNA-treated group (Figure 6C).

### STAP2 enhances the STAT3/SOCS3 cascade in CD8^+^ T cells after antigenic stimulation

**Figure 7:**
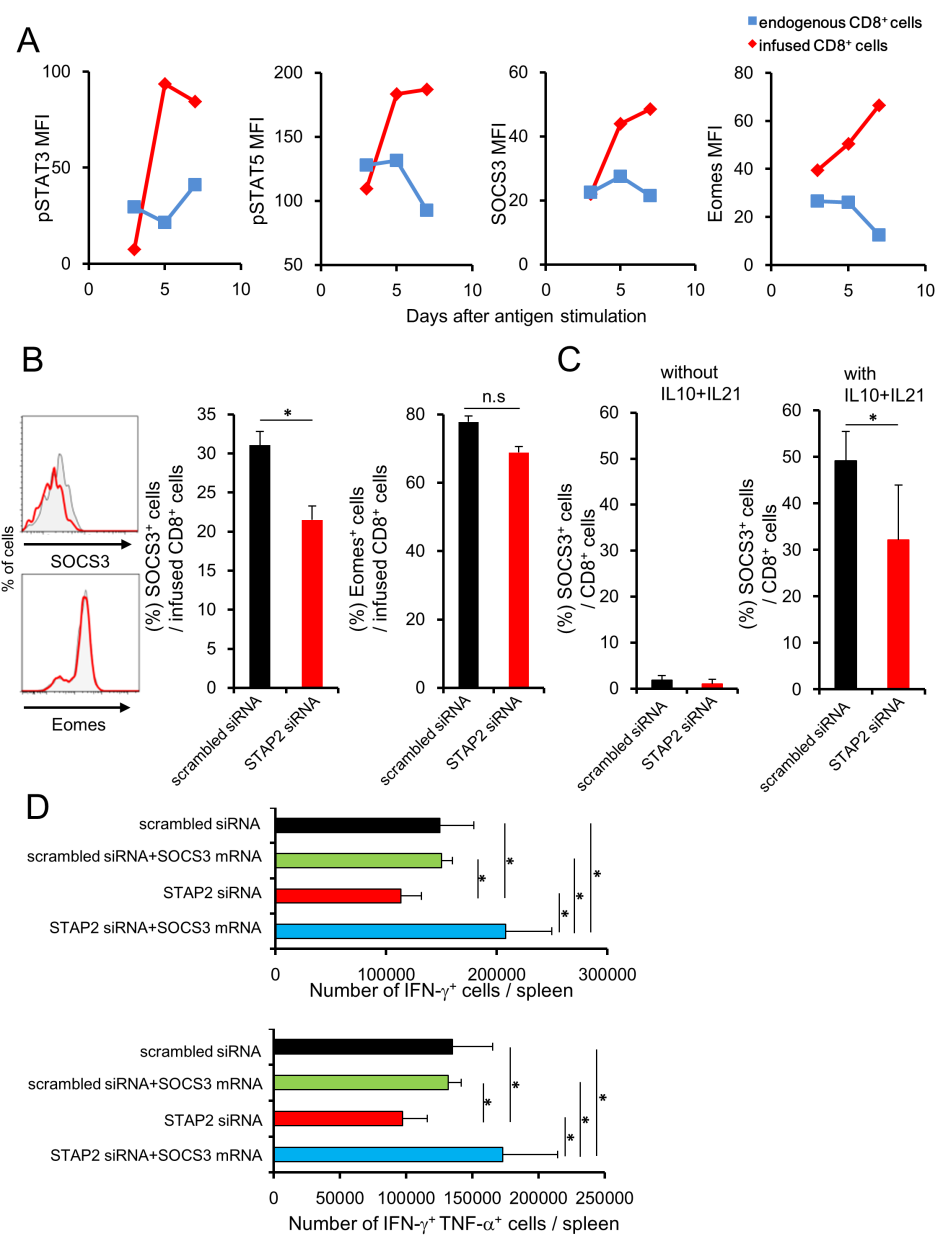
Attenuation of STAT3/SOCS3 cascade in antigen-stimulated CD8^+^ T cells by STAP2 knockdown BALB/c mice were infused with DUC18 CD8^+^ T cells and then immunized with plasmids encoding mERK2 by gene gun. **A**. At the indicated days, pSTAT3, pSTAT5, SOCS3, and Eomes expression in the splenocytes of DUC18 CD8^+^ T or in endogenous CD8^+^ T cells was measured by flow cytometry. (B, C) BALB/c mice were infused with siRNA-transduced DUC18 CD8^+^ cells and then immunized with plasmids encoding mERK2 by the gene gun. **B**. At 4 days after immunization, SOCS3 and Eomes expression in the infused cells was measured by flow cytometry. **C**. SOCS3 and Eomes expression in infused cells was measured after IL-10 and IL-21 stimulation. **D**. BALB/c mice were infused with the indicated siRNA- and/or mRNA-transduced DUC18 CD8^+^ T cells and then immunized with plasmids encoding mERK2 by the gene gun. At 44 days after injection, CMS5a were inoculated subcutaneously into these mice and IFN-γ and TNF-α production was examined. The results are representative of two to four experiments. Data are expressed as the mean ± SD. *p < 0.05 is considered significant. n.s. = not significant.

We analyzed the relationship between STAP2 and STATs signaling pathway during memory cell differentiation to further confirm the importance of STAP2 in the maintenance of memory CTL function. Since STAT3 and STAT5 signals have been reported to regulate the memory T cell differentiation, [5, 14] we initially investigated the kinetics of phosphorylated (p) STAT3 and pSTAT5 expressions in DUC18 CD8^+^ T cells primed with mERK2-encoded DNA in infused BALB/c mice. Enhanced pSTAT3 and pSTAT5 expressions were observed in the primed DUC18 CD8^+^ T cells in correlation with the elevation of SOCS3 and Eomes expressions, both of which have been reported as downstream molecules of STATs (Figure 7A).[5, 8, 14] In addition, STAP2 siRNA-introduced DUC18 CD8^+^ splenic T cells showed significantly lower SOCS3 expression than their scrambled siRNA-transduced counterparts *ex vivo*, even though there was no difference in Eomes expression between STAP2 and control siRNA-transfected cells (Figure 7B). IL-21 and IL-10 have been known to regulate memory T cell differentiation through STAT3/SOCS3 signaling, indicating that these cytokines are located upstream of STAT3/SOCS3 signaling.[14] At 2 days after vaccination of BALB/c mice infused with siRNA-introduced DUC18 CD8^+^ T cells, the infused cells were collected and treated *in vitro* with recombinant (r) IL-10 and rIL-21 for 8 h. Increment of SOCS3 expression by treatment with IL-10 and IL-21 was significantly abrogated by STAP2 KD (Figure 7C). To evaluate the effects of SOCS3 on the maintenance of CTL function by STAP2, we established SOCS3 mRNA-transfected DUC18 CD8^+^ T cells. As shown in Figure 7D, SOCS3 overexpression rescued the dysfunction of STAP2 siRNA-transduced memory CD8^+^ T cells, as expected. Notably, enhancement of the secondary memory CD8^+^ T cell response was not observed when scrambled siRNA was co-transfected, clearly indicating the synergistic effect of STAP2 KD and SOCS3 overexpression.

## DISCUSSION

By STAP2 knockdown in antigen-experienced CD8^+^ T cells, we demonstrated that STAP2 was not only dispensable during the effector phase but was also essential for the formation of functional memory CD8^+^ T cells and their recall responses in a T cell-intrinsic manner. Mechanistically, STAP2 deficiency resulted in impaired cytotoxicity and cytokine production from memory CTLs in association with attenuation of STAT3 signaling. In addition, STAP2 loss caused unresponsiveness of both IL-10- and IL-21-mediated STAT3 signals and downregulation of SOCS3 expression. These findings provide a novel insight in functional memory T cell formation, and suggest that artificial modification of STAP2 expression can be a potent strategy for improving the quality of memory T cell responses.

Our studies also clarified a crucial role of STAT3 signaling in the determination of memory CD8^+^ T cell differentiation. A study from another group has shown that STAT3-disrupted CD8^+^ T cells differentiate into dysfunctional KLRG^+^ IL-7R^−^ long-lived memory phenotype rather than the functional KLRG^−^ IL7R^+^ memory phenotype.[14] Likewise, we observed a decrease in long-lived memory CTL function and a decrease in the number of KLRG^−^ IL-7R^+^ memory CD8^+^ T cells in association with elevated KLRG^+^ population by attenuation of STAT3/SOCS3 signals after siRNA-mediated STAP2 KD (Supplementary Figure S4). Since reduced STAT3 signaling was seen soon after STAP2 KD, STAP2 acts a crucial role in differentiation of T cells into functional memory phenotype at an early stage after antigen stimulation.

STAP2 has been reported to elicit various functions (adhesion, chemotaxis and cell survival [19–23]). These functions seem to be irrelevant to the maintenance and differentiation of functional memory T cells as demonstrated in this study because siRNA-mediated STAP2 KD did not affect on the number of infused T cells on d-4 or d-44 after infusion (Supplemental Figure S2 and S3) nevertheless fluctuation of degree of cell-cell contact, chemotactic condition and viability directly associates with the total number of cells in the secondary lymphoid organ.

Consistent with previous reports from other groups using STAT3 KO mice, our study showed elevation of KLRG expression in part of STAP2 KD long-lived memory CD8^+^ T cells. This indicates a shift to terminal effector phenotype because it is known that downregulation of SOCS3 in STAT3 KO cells elevates the sensitivity of STAT4-mediated pro-inflammatory cytokines, such as interleukin-12,[29] which enhance effector cell differentiation to the terminal phenotype. KLRG^+^ terminal effector cells are known to exhibit strong cytotoxicity. In contrast, tumoricidal activity of long-lived KLRG^+^ memory CTL was scarcely observed in this study, considering the correlation of T cell senescence characterized by low proliferative potential after terminal differentiation with memory T cell dysfunction. Memory T cells are classified into Tscm, Tcm, and Tem. It has been shown that the Wnt-beta-catenin pathway is essential for Tscm differentiation, and that this signal arrests CD8^+^ T cell development in an early differentiation phase and promotes the generation of functional Tscm with tumoricidal activity.[6] In this study, STAP2 loss affected the memory T cells in the spleen, but not in the LNs.

Currently, although It is difficult to clearly distinguish Tem and Tcm, spleen and LN seems to be reservoir for Tem and Tcm by difference of expression level of LN-homing molecules as reported previously [30], respectively. STAP2 may be a rare molecule that operates in Tem, while precise analysis remains to be elucidated. Taken together, STAP2 acts as a key intracellular molecule in the process of the memory T cell differentiation probably including tissue residential memory T cells,[31], [32] which have received extensive attention in the medical areas of allergy, autoimmune disorders, infectious diseases, and cancer.

To improve the efficacy of adoptive cell therapy for patients with cancer, the cytokine status of the CD8^+^ T cell expansion was examined rather than the quality required for the maintenance and generation of long-lived memory CD8^+^ T cells. Our results suggest the potential to provide high-quality memory CTLs by regulating STAP2 expression after antigen stimulation.

## MATERIALS AND METHODS

### Mice

Female BALB/c mice were obtained from SLC Japan and used at 6 to 12 weeks of age. DUC18 mice, transgenic for TCRα/β reactive with H-2K^d^-restricted mutated (m) ERK2 (136-144 aa), were established as described previously[27]. CD90.1-congenic BALB/c mice were kindly provided by Dr. S. Sakaguchi (Osaka University, Japan). DUC18 mice and CD90.1-congenic mice were mated to obtain DUC18/CD90.1 F1 animal. All animal experiments were approved by the Institute of Laboratory animals at Mie University (#24-14). All experiments were conducted in accordance with the institute guidelines.

### Measuring tumor growth

A 3-methylcholanthrene-induced sarcoma cell line CMS5a (BALB/c origin) expresses mERK2 protein as the mutated antigen.[26] CT26 is a colon epithelial tumor cell line derived from treatment with *N*-nitroso-*N*-methylurethane in BALB/c mice.[33] CT26 cells expressing a human cancer/testis antigen NY-ESO-1 protein (CT26-NY-ESO-1) were established as described previously.[25] Mice were inoculated subcutaneously in the right hind flank with 1 × 10^6^ CMS5a cells or CT26-NY-ESO-1 cells and monitored twice or thrice per week [25]. Tumor volumes were calculated as follows: tumor volume (mm^3^) = 0.5 x length (mm) x [width (mm)]^2^.

### Peptide and DNA immunization

NY-ESO-1 81-88 peptide (100 μg/mouse) emulsified in Montanide™ (incomplete Freund's adjuvant) was injected subcutaneously in the right flank of mice on a weekly basis. Fifty μg CpG ODN 1668 was subcutaneously injected. Gold particles coated with 1 μg of plasmid DNA were prepared and delivered into the shaved abdominal skin of BALB/c mice by a Helios Gene-Gun System (BioRad) at a helium discharge pressure at 350-400 psi, as described previously.[24]

### Microarray analysis of NY-ESO-1-specific CD8^+^ T cell subpopulation

Draining lymph-node cells from 40 to 60 mice, obtained at 7 days after vaccination were pooled and stained with PE-labeled NY-ESO-1_81-88_-H-2D^d^ tetramers (TCMetrix, Epalinges, Switzerland) for 10 min at 37°C before additional staining with allophycocyanin (APC)-CD8-specific mAb for 15 min at 4°C. After washing, NY-ESO-1_81-88_-H-2D^d^ tetramers^+^ CD8^+^ T cells were sorted using a FACSAria system (BD Biosciences). cDNA was prepared from separated CD8^+^ T cells and hybridized to Affymetrix GeneChip Mouse Genome 430 2.0 arrays.

### Quantitative real-time PCR

Total RNA isolated using the RNeasy micro kit (QIAGEN) was subjected to reverse transcription reaction with the QuantiTect Reverse Transcription Kit (QIAGEN). RT-PCR was performed to quantify mRNA levels of specific genes using TaqMan^®^ Gene Expression Assays (ABI). To quantify STAP2 expression, we used GAPDH mRNA as an endogenous control. ∆∆Ct = ∆Ct (STAP2- GAPDH: each sample)- ∆Ct (STAP2- GAPDH: reference sample), and fold change = 2^−∆∆CT^. ∆CT in the peptide vaccine group, the IL7R(-) CD25(-) group, and the IL7R Low group were used as reference samples for Figure 1D, 1G, and 1H, respectively. Each experiment was performed in triplicate and confirmed by at least three biological replicates.

### Isolation of CD8^+^ cell subpopulations

CD8^+^ T cells from spleen cell suspensions were isolated negatively using magnetic microbeads (Miltenyi Biotec) according to manufacturer's instructions. To isolate IL-7 receptor (R)^+^ cells, the antigen-stimulated cells were stained with fluorescein isothiocyanate (FITC)-anti-CD8, phycoerythrin (PE)-anti-IL-7R, and APC-CD25 or peridinin chlorophyll protein complex with cyanin-5.5 (PerCP-Cy5.5)-anti-CD90.1 monoclonal antibodies (mAbs) and were then sorted on the FACSAria system (BD Biosciences).

### Flow cytometric analysis

Cell suspensions from inguinal LNs or the spleen were stained with surface marker-specific mAbs in PBS with 2% fetal bovine serum for 15 min at 4°C and analyzed on the FACSCanto II (BD Biosciences). For intracellular cytokine staining, cells stimulated with the relevant peptide or the control peptide (30 min, 37°C) were incubated for 6 h with GolgiPlug (BD Biosciences). After permeabilization and fixation using a Cytofix/Cytoperm Kit according to the manufacturer's instructions (BD Biosciences), the obtained cells were stained with FITC-conjugated surface marker-specific, APC-conjugated anti-IFN-γ, and PE-conjugated anti-TNF-α mAbs. For staining of intracellular signaling molecules, cells were fixed and permeabilized with cytofix and phosflow buffer I and III (BD Biosciences), respectively, and then stained with specific Abs. After washing, the stained cells were subjected to analysis on FACSCanto II (BD Biosciences) and data were analyzed with FloJo software (Tree Star).

### Antibodies and reagents

Anti-CD8 (53-6.7), anti-CD90.1 (OX-7), anti-killer cell lectin-like receptor KLRG (2F1/KLRG1), purified anti-CD127 (A7R34), and anti-CD25 (PC61) mAbs were purchased from Biolegend. Anti-IFN-γ (XMG1.2) and anti-TNF-α (MP6-XT22) mAbs were obtained from eBioscience. Phosphorylated (p)STAT3-, pSTAT5-, and Eomes-specific mAbs were purchased from BD Bioscience. SOCS3-specific mAb was obtained from Cell Signaling Technologies. Synthetic NY-ESO-1 81-88 (RGPESRLL) and mERK2 136-144 peptides (QYIHSANVL) were obtained from Sigma Co. Ltd. All siRNAs were designed and synthesized by Sigma-Aldrich (MISSION^®^ siRNA ). CpG ODN 1668 (5′-TCCATGACGTTCCTGATGCT-3′) were synthesized by Hokkaido System Science (Sapporo, Japan).

### Electroporation for cultured and naïve T cells

For siRNA transfection, CD8^+^ T cells stimulated with mERK2 peptide were suspended in phosphate-buffered saline (PBS), and then resuspended in OPTI-MEM (Invitrogen, Carlsbad, CA). siRNA was mixed with the cell suspension at a density of more than 2 × 10^6^ cells per 100 μl, transferred into a 2-mm gap cuvette (Genetronics Inc., San Diego, CA), and subjected to electroporation using the BTX ECM 830 square-wave electroporator (Genetronics Inc.). After electroporation, the cells were suspended in 2 ml of complete RPMI-1640 medium, and immediately cultured in one well of a 24-well plate for 1 h at 37°C until infusion.

Nucleofector electroporation system (Lonza) and Mouse primary T cell Nucleofector kit (Lonza) were used to transduce siRNA into naïve T cells. Purified CD8^+^ cells were suspended in the Nucleofector solution with siRNA or control siRNA at a final density of 2 × 10^6^ cells per 100 μl, and immediately transferred into an Amaxa cuvette and subjected to electroporation. After electroporation, the cells were suspended in complete Mouse T Cell Nucleofector Medium, and then cultured in one well of a 24-well plate for 1 h at 37 °C until infusion.

### *In vitro* transcription for mRNA preparation

Before *in vitro* mRNA synthesis, the plasmids were linearized with appropriate restriction enzymes. *In vitro* transcription was performed with T7 polymerase according to the instructions provided (mMESSAGE mMACHINE T7 Kit; Ambion). The *in vitro*-transcribed RNA was then polyadenylated using Poly (A) Polymerase (Poly (A) Tailing Kit; Ambion) according to the manufacturer's instructions. The resulting capped and tailed RNA was resuspended in PBS and stored at -80°C until used for transfection.

### Proliferation analysis

Spleen cells labeled with CFSE in PBS for 5 min at 37 °C were stimulated with the relevant peptide or the control peptide for 3 days. The obtained cells were subjected to analysis on FACSCanto II (BD Biosciences) and data were analyzed with FlowJo software (Tree Star).

### Statistical analysis

The results for *in vivo* tumor growth were analyzed using the Steel-Dwass test for multiple comparisons among experimental groups. The remaining data were analyzed using Student's *t*-test or one-way ANOVA. *p* < 0.05 was considered as significant.

## SUPPLEMENTARY MATERIALS FIGURES


