# Supplementary material for: Signal-transducing adaptor protein-2 promotes generation of functional long-term memory CD8+ T cells by preventing terminal effector differentiation
Source: Oncotarget. 2017 Feb 16;8(19):30766–80. doi: 10.18632/oncotarget.15403 (PMC5458166; doi:10.18632/oncotarget.15403)
Supplement: Supplementary file 1 [file oncotarget-08-30766-s001.pdf]

Signal-transducing adaptor protein-2 promotes generation of functional long-term memory CD8^{+} T cells by preventing terminal effector differentiation

Supplementary Material

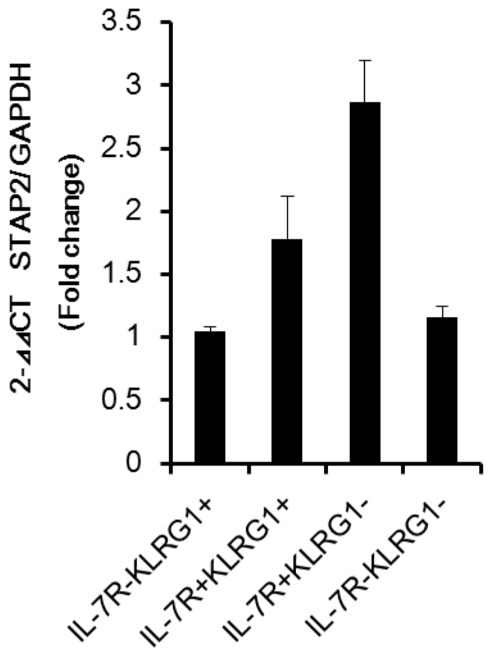

Supplementary Figure S1: Splenocytes derived from DUC18 mice were stimulated with mERK2 peptide for 3 days and then cultured in rIL-2 (10 IU/ml)-containing medium for 4 days. STAP2 expression in cultured cells was measured by RT-PCR.

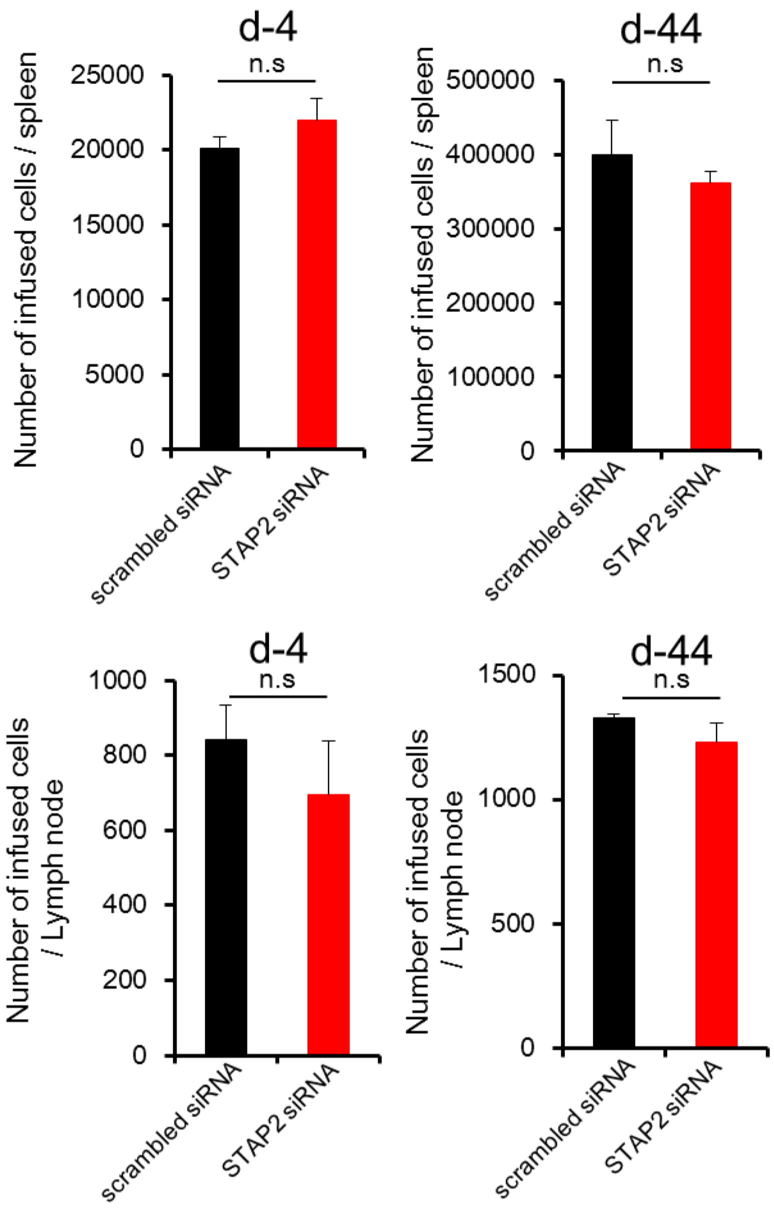

Supplementary Figure S2: Splenocytes derived from DUC18 mice were stimulated with mERK2 peptide for 3 days, transduced with STAP2-specific siRNA or scrambled control siRNA by electroporation, and injected into BALB/c mice. At 4 or 44 days after infusion, the number of infused cells was examined. The results are representative of two to four experiments. Data are expressed as the mean ± SD. *p < 0.05 is considered significant.

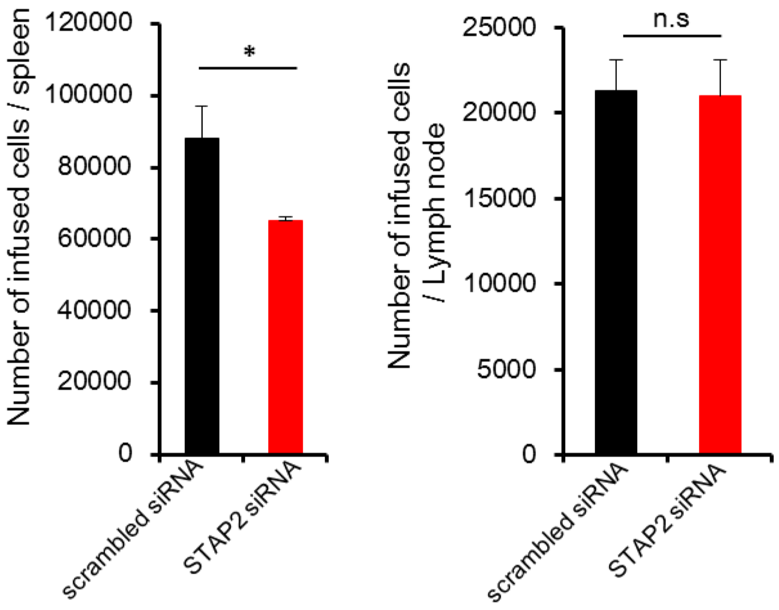

Supplementary Figure S3: Naïve CD8^{+} cells derived from DUC18 mice were transduced with STAP2-specific siRNA or scrambled control siRNA by electroporation, and injected into BALB/c mice. These BALB/c mice were then immunized with plasmids encoding mERK2 using a gene gun. At 44 days after vaccination, the number of infused cells was examined. The results are representative of two to four experiments. Data are expressed as the mean ± SD. *p < 0.05 is considered significant.

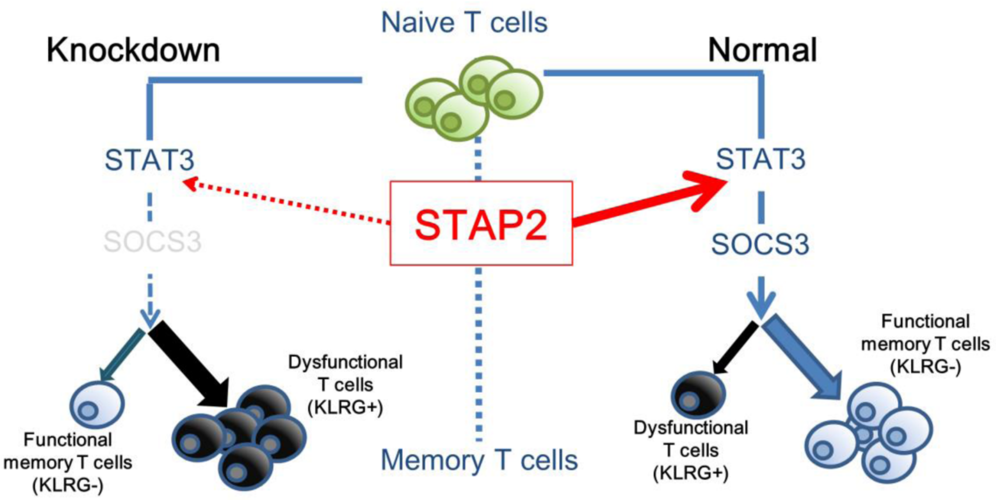

Supplementary Figure S4: In normal T cells, enhancement of STAT3/SOCS3 signals by STAP2 leads the generation of functional memory CTLs. In contrast, STAP2 KD T cells result in the differentiation of dysfunctional CTLs in association with the attenuated STAT3/ SOCS3 signals.
